# The reproductive strategy of a pollinator-limited Himalayan plant, *Incarvillea mairei* (Bignoniaceae)

**DOI:** 10.1186/1471-2229-13-195

**Published:** 2013-12-01

**Authors:** Honglian Ai, Wei Zhou, Kun Xu, Hong Wang, Dezhu Li

**Affiliations:** 1 Key Laboratory for Plant Biodiversity and Biogeography of East Asia, Kunming Institute of Botany, Chinese Academy of Sciences, Kunming, China; 2 Plant Germplasm and Genomics Center, Germplasm Bank of Wild Species, Kunming Institute of Botany, Chinese Academy of Sciences, Kunming, China; 3 Lijiang Alpine Botanic Garden, Kunming Institute of Botany, Chinese Academy of Sciences, Lijiang, China

**Keywords:** Himalaya-Hengduan mountains region, *Incarvillea mairei*, Plant-pollinator interaction, Pollinator limitation, Sensitive stigma

## Abstract

**Background:**

Plants may adapt to alpine habitats by specialization in the reproductive strategy and functional aspects of their flowers and pollination systems. Alpine habitats reduce the opportunities for cross-pollination in a relatively high proportion of alpine plant species, and self-pollination may be favored in these adverse conditions. Here, we investigated the mating system and pollination of *Incarvillea mairei*, a perennial Himalayan herb typically found at altitudes between 3000 and 4500 m.

**Results:**

Analyses of floral morphology, observation of plant-pollinator interactions, and hand pollination experiments were conducted in three natural populations. Outcrossing rates and effective numbers of pollen donors were assessed in 45 open-pollinated families by using progeny analysis based on seven microsatellite markers. *Incarvillea mairei* displayed a set of apparently specialized floral traits, the stigma is sensitive to touch and close immediately and its reopening allows a second opportunity for the receipt of pollen. The species is fully self-compatible but employs a predominantly outcrossing mating system according to parentage analysis (t_m_ > 0.9). Fruit set was low (26.3%), whereas seed set was high (67.2%), indicating that this species suffers pollinator limitation. Its main effective pollinator was *Halictus* sp., and visitation frequency was low.

**Conclusions:**

Floral features such as having a sensitive stigma and anther-prongs, in conjunction with pollinator behavior, function together contributing to a set of unique reproductive adaptations that enhance outcrossing success. The increased floral longevity and high pollination efficiency operated as compensatory mechanisms to counteract low pollinator visitation frequency.

## Background

Reproductive success in stressful or variable environments in which pollination may be uncertain is a common problem that confronts many lineages of seed plants. Alpine environments are one such habitat in which short growing seasons, low densities of insect pollinators, and harsh weather during the growing season work against effective pollination and seed set [1-4]. Consequently, plants with selfing mechanism may be favored in these harsh conditions, because their reproduction does not depend on pollinators [5-7]. Indeed, it has been demonstrated that selfing rates increase with latitude and altitude [8-10]. Contrary to this trend, Wirth et al. [11] revealed lower selfing rate at higher altitudes in *Eritrichium nanum* (Boraginaceae), an alpine cushion plant in the Swiss Alps, although this may be explained by unfavourable weather conditions at the beginning of the growing season. The Himalaya-Hengduan mountains region hosts a variety of alpine habitats and a plethora of interesting alpine plant species [12], yet few studies have addressed the pollination biology of these plants [13].

Pollinator limitation is a commonly encountered problem for outcrossing species in alpine habitats and is expected to increase at higher altitudes [14-17]. However, these species have evolved many reproductive strategies to cope with unfavourable pollination conditions [18]. For example, prolonged floral longevity can compensate for low pollinator visitations [1,17,19,20]. Floral traits also substantially influence pollination success; for instance, the herkogamous flower is expected to reduce self-fertilization allowing more opportunity for outcrossing [21,22]. Individual species may use complex variations of these strategies to ensure reproductive success [23-26].

The genus *Incarvillea* is a predominantly Himalayan, high altitude genus of herbaceous plants in Bignoniaceae, a family of mostly tropical woody plants. This genus contains 18 species, 16 of which occur in the alpine areas of the Himalaya-Hengduan mountains region [27]. *Incarvillea* species have specialized floral structures with obvious herkogamy, often including a bilobed sensitive stigma and anther appendages [28,29]. As early as 1921, Cutting [28] observed *I. delavayi*, a Himalayan species cultivated in European gardens, and speculated that the sensitive stigma and anther appendages might assist in cross-pollination. Recently, the anther appendages of *I. arguta*, a low-altitude species (1300-2700 m), have been found to play a role in triggering its pollen-dispensing mechanism [29]. In addition, *I. sinensis* of Inner Mongolia in northern China has developed a strategy of delayed self-pollination involving wind-driven corolla abscission [30]. The reproductive strategy of the alpine species of *Incarvillea* endemic to the Himalaya-Hengduan mountains region have not been revealed, consistent with the general paucity of information on the reproductive biology of the flora in that region.

Here, we investigated the reproductive biology of *I. mairei*, a Himalayan herb typically found at altitudes between 3000 and 4500 m. The purpose of our study was to determine the reproductive strategy of *I. mairei*. Specifically, we address the following questions: (1) What is the breeding system of *I. mairei*? (2) Are fruit set and/or seed set pollen-limited? (3) What is the outcrossing rate for this species evaluated by microsatellite markers analyses? (4) How do floral traits associate with pollinator influence its reproductive success?

## Methods

### Study species and sites

**Figure 1:**
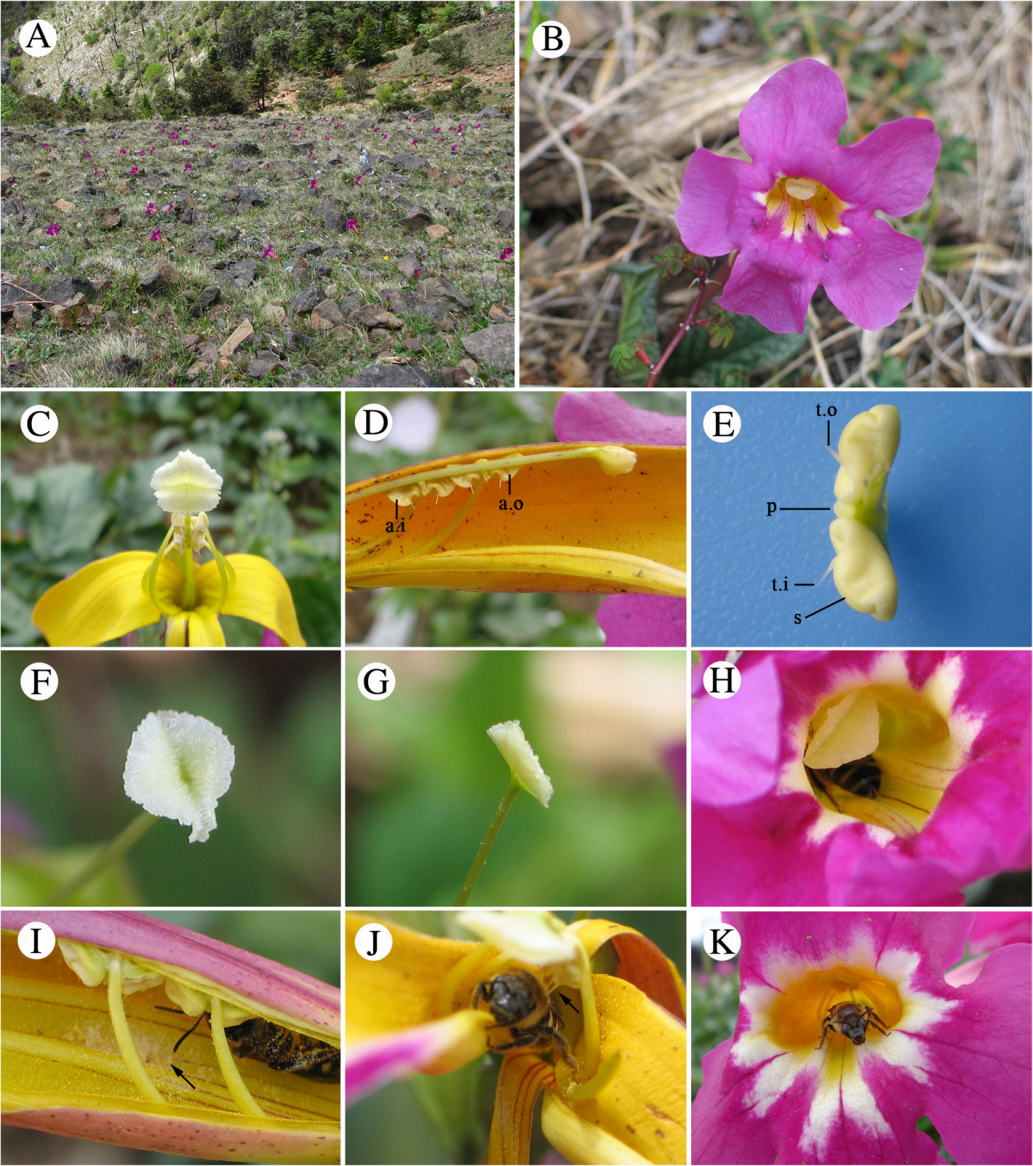
**Flower structures of *Incarvillea mairei *and pollinator behavior. (A)** Rocky meadow habitat of population GML. **(B)** Mature flower. **(C)**The large, bilobed sensitive stigma and four anthers. **(D)** Longitudinal section of flower. **(E)** Anther appendages (attached oppositely to each lobe). **(F)** Opened stigma. **(G)** Closed Stigma. **(H)** Halictid bee touching the stigma. **(I)** Halictid bee is pressing the anther-thorns (arrow indicating released pollen). **(J)** Halictid bee exiting flower. **(K)** After bee has exited, the stigma remained closed. a.i: inner anther; a.o: outer anther; t.o: outer anther-thorn; t.i: inner anther-thorn; s: slit in anther-lobe; p: pad.

*Incarvillea mairei* mainly grows on rocky, grassy slopes and meadows (Figure 1A). Flowering occurs in early spring after snowmelt. This plant is a stemless perennial herb with two to five pairs of leaflets. The inflorescences are racemose, and individual plants produce one to four flowers. Flowers are showy and each possesses a large, sensitive stigma composed of two lobes positioned in front of the anthers (Figures 1B-D). The four stamens are didynamous and epipetalous, with anthers pressed closely against the style (Figures 1C and D). Each anther bears two stiff prongs in opposing directions, one on each lobe, connected to a protuberant pad (Figure 1D and E). The two lobes of the stigma begin to open following the initiation of anthesis (Figure 1F) and close immediately following detection of touch stimuli (Figure 1G).

From May to August, fieldwork was conducted on the Yulong Snow Mountain in Lijiang county, northwestern Yunnan, China for three consecutive years (2007–2009). The fieldwork permit was obtained from Lijiang Alpine Botanic Garden, Kunming Institute of Botany, CAS. *I. mairei* is a locally rare species in the study sites, and the frequency of known populations is very low. Only three natural populations were selected: (1) Ganheba, “GHB” (geographical coordinates 27°03' N, 100°15' E; 3190 m); (2) Haligu, “HLG” (27°00' N, 100°11' E; 3290 m); and (3) Guomeiluo, “GML” (27°05' N, 100°13' E; 4000 m). These populations were separated by 5–20 km. For genetic progeny analysis, the seeds of each field-collected fruit were sown separately in 2008 in a greenhouse at the Kunming Botanical Garden, Kunming, Yunnan, China (25°08'42″ N, 102°44'31″ E).

### Stigma movements

Before the start of the flowering season in the population HLG, thirty plants were selected and observed daily throughout the flowering season. The duration of anthesis, anther dehiscence, and opening and closure of stigmas were recorded. The separate effects of touch and pollen on stigma closure were tested in the population HLG. First, chosen flowers were bagged to exclude insects. Two treatments were applied to separate groups of open stigmas: (1) mechanical touch (N = 30); and (2) hand cross-pollination (N = 30). For the latter treatment, pollen was gently sprinkled onto the stigma to prevent the immediate closure caused by touch. In each treatment, records were made on whether stigma closure and reopening occurred, and if so, the timing of the events.

### Floral longevity

Floral longevity was recorded in the GHB and GML populations. One floral bud on each of 20 different plants per population was marked and covered with insect-excluding netting in otherwise natural conditions. Flower survival was recorded every other day until senescence occurred.

### Pollinator observations and pollination experiments

Insect observations were conducted continuously during peak flowering, from 10:00 to 15:00 for 4 d in GHB and HLG of 2007 and 2008, and for 3 d in GML in 2009. Following Bittencourt and Semir [31], stigma closure was considered to be an indication that flowers had been visited and pollinated. Pollinators and their foraging behaviors were observed and photographed. Insect specimens were identified and preserved in the insect collection of the Kunming Institute of Botany, CAS.

Six pollination treatments were conducted in HLG in 2007 and 2008: (1) natural pollination: flowers were not manipulated (N = 170); (2) autonomous apomixis: buds were bagged and anthers were removed before flowering (N = 20); (3) autonomous self-pollination: buds were bagged throughout their flowering period (N = 20); (4) hand self-pollination: bagged flowers were hand pollinated with pollen from the same flower (N = 21); (5) hand cross-pollination: bagged flowers were emasculated before anthesis and pollinated with pollen from plants growing 5–10 m away (N = 28); and (6) supplementary pollination: flowers were exposed to natural pollination for two days, and then pollinated with cross-pollen (N = 25). To further examine the effect of pollen limitation on seed production, supplementary pollination was performed in GML and GHB in 2008 (N = 25, respectively). All bags were removed following floral wilting. The numbers of fruits were recorded after two weeks and fruit set was estimated as the proportion of flowers setting fruits. Mature fruits were collected later, and the numbers of mature seeds and aborted or unfertilized ovules were determined to assess seed set.

### Genetic sampling, DNA extraction and genotyping

Thirty individuals per population were randomly chosen. Leaf tissue was sampled and preserved in silica gel. From each population, fifteen naturally pollinated fruits (one fruit per plant) were harvested and matured seeds were collected from each fruit. The seeds from each fruit were sown separately in a greenhouse at the Kunming Botanical Garden. Ten seedlings per family were randomly selected (150 individuals/population or 450 individuals as a whole) and used for genetic analyses.

Genomic DNA was extracted using a modified cetyl trimethyl ammonium bromide (CTAB) protocol [32]. Seven primer pairs: IM6, IM7, IM26, IM48, IM73, IM75 and IM78 were used to genotype all adult individuals as well as the open-pollinated progeny, using *I. mairei* microsatellite loci [33]. Forward primers were labeled with a fluorescent dye (HEX, TAM or FAM). The PCR reactions were performed in 15 μl reaction volumes containing 30-50 ng genomic DNA, 0.6 μM of each primer, and 7.5 μl 2 × Taq PCR Master Mix (Tiangen; 0.1U Taq Polymerase/μl, 0.5 mM dNTP each, 20 mM Tris–HCl (pH 8.3), 100 mM KCl, 3 mM MgCl_2_). PCR amplifications were conducted under the following conditions: 94°C for 3 min, 30–36 cycles of 94°C for 30s, primer-specific annealing temperature for 30s, and 72°C for 1 min, and a final extension step at 72°C for 8 min. The PCR reactions and amplifications were performed on a Gene Amp 9700 DNA Thermal Cycler (Applied Biosystems, CA, USA), and the PCR products were examined using an ABI PRISM 3100 automated sequencer. The genotypes were analyzed by Gene Mapper version 3.2.

### Statistical analyses

All analyses were performed in SPSS 13.0 (SPSS, Chicago, IL, USA) for Windows. All parameter estimates were calculated as mean ± SE (Standard Error) values. Two-way ANOVAs were used to compare the flower longevity between lower-altitude (GHB) and higher-altitude population (GML) under natural pollination and bagged conditions. The fruit set and seed set were analysed using MANOVA with pollination treatment, population and year as fixed effects. Because fruit set and seed set may be inter-correlated, this MANOVA was used initially to control for type I error [34]. A significant MANOVA was followed by univariate ANOVA, using the Bonferroni method for multiple comparisons. None of the interactions between treatment and population or year were significant (*P* > 0.05) in both cases, these interactions were removed from the final models.

Summary statistics of the microsatellite loci calculated, including number of alleles per loci (*A*), observed and (*H*_*O*_) expected heterozygosities (*H*_*E*_) under Hardy–Weinberg equilibrium, and fixation index (F) and inbreeding coefficient (F_IS_) based on the adult populations. Analyses were performed using the software FSTAT 2.9.3.2 [35] and GenAlEx version 6.2 [36].

Genetic data were analyzed using the MLTR version 3.4 program [37]. In this analysis, we used the genotypes of progenies to estimate the mating system parameters of the maternal parent in the field using the Expectation-Maximization method [37]. Outcrossing rate estimates were calculated using multilocus and average single locus estimates (respectively *t*_m_ and *t*_s_) based on Ritland’s mixed mating model [38]. The comparison between the two outcrossing estimates (*t*_m_ – *t*_s_) provides an estimation of biparental inbreeding, i.e., inbreeding as a result of outcrossing with related individuals [39]. All mating system parameters were estimated at population level. Standard errors (SE) and 95% confidence intervals (CI) for the mating system parameter estimates were obtained from 1000 bootstrap replicates of the data. Bootstrap estimates of *t*_m_ were compared to 1, corresponding to strict outcrossing, and *t*_m_ – *t*_s_ estimates were compared to zero.

The correlated paternity presented here is the assignment procedure of the sibling relationship in each fruit. The assignment of the sibship can estimate the number of pollen donors and their offspring. The paternity correlation (*C*_p_) is inversely related to the number of outcross parents by *C*_p_ =1/*N*_ep_, where *N*_ep_ is the effective number of pollen donors [39]. To confirm the reliability of the assignment procedure, two estimators of correlated paternity based on the following approaches were conducted: *r*_p_ was estimated with the outcrossing rate fixed at 94%, and *F* and *r*_t_ were fixed at zero by Ritland’s mixed mating system model. 2Φ_FT_ was also estimated by TWOGENER analysis.

## Results

### Stigma movements

The two stigma lobes began to open immediately following the initiation of anthesis and remained open until the flower senescence. Closure in response to touch was rapid, taking place in only 6.86 ± 3.06 s (SE, N = 30), while closure in response to pollen deposition required 0.5-1.5 h (N = 30). If low numbers of pollen grains were received, most stigmas (>80%) reopened after 8–15 h, whereas the stigmas that closed following touch without pollen reopened after 19.15 ± 2.28 min (SE, N = 30). Under natural conditions, only 26% (N = 100) of the stigmas were found to be closed in the population HLG, but most stigmas in hand-pollinated flowers (92.5%, N = 80) remained closed for the remainder of floral lifetime. Flowers that had been bagged to exclude pollinators were intact, with no pollen released and the bi-lobed stigmas remaining open until flowers faded.

### Floral longevity

Anthesis lasted for 8.25 ± 1.69 days (ranging from 6 to 12) (SE, N = 40). Duration of flowering in GHB was 7.40 ± 1.23 and 9.45 ± 1.36 (SE, N = 20) days under natural conditions and bagged treatment, respectively, while in GML natural and bagged flowers bloomed for 9.10 ± 1.68 and 13.5 ± 1.93 (SE, N = 20) days, respectively. The effects of altitude and treatment on floral longevity were significant (*F* = 66.634, *P* < 0.001, and *F* = 83.845, *P* <0.001, respectively).

### Pollinator observations

A small number of insects were observed in the three study sites. In the higher-altitude GML population, only three bumblebee queens were observed visiting flowers of *I. mairei*. Frequencies of bumblebee visits were too low to allow reliable quantification. Stigma closure seems to be an indication that flowers had been visited and pollinated [31]. In the 100 flowers, only in 23, 26, and 27 were the stigma found closed in GML, HLG, and GHB, respectively. We recorded the behavior of *Halictus* bees found foraging on and pollinating *I. mairei* in the lower-altitude GHB and HLG populations, they were observed to be effective visitor, where the average visiting frequencies were 0.007 visits/flower/hr and 0.005 visits/flower/hr, respectively. Pollination proceeded in detail as follows: First, the halictid bee accessed the flower following the two marked ridges running down the anterior of corolla tube, and made contact with the stigma lobes. This caused pollen grains to be removed from the dorsal side of the pollinator’s body (Figure 1H) and the stigma closed immediately afterward. More precisely, upon entering the flower, the bee’s back pressed a pair of “trigger” appendages of the outer anther lobes, causing the anther clefts to close tightly and prevent pollen release. Subsequently, the reverse pressure onto a different pair of anther appendages caused the inner anther lobes to open and discharge pollen onto the dorsal surface of the bee (Figure 1I). The sequence occurred in reverse as the bee exited the flower, causing the outer pair of anther-lobes to open and shed additional pollen grains (Figure 1J). The stigma remained closed (Figure 1K) following departure of the bee.

### Breeding system

**Figure 2:**
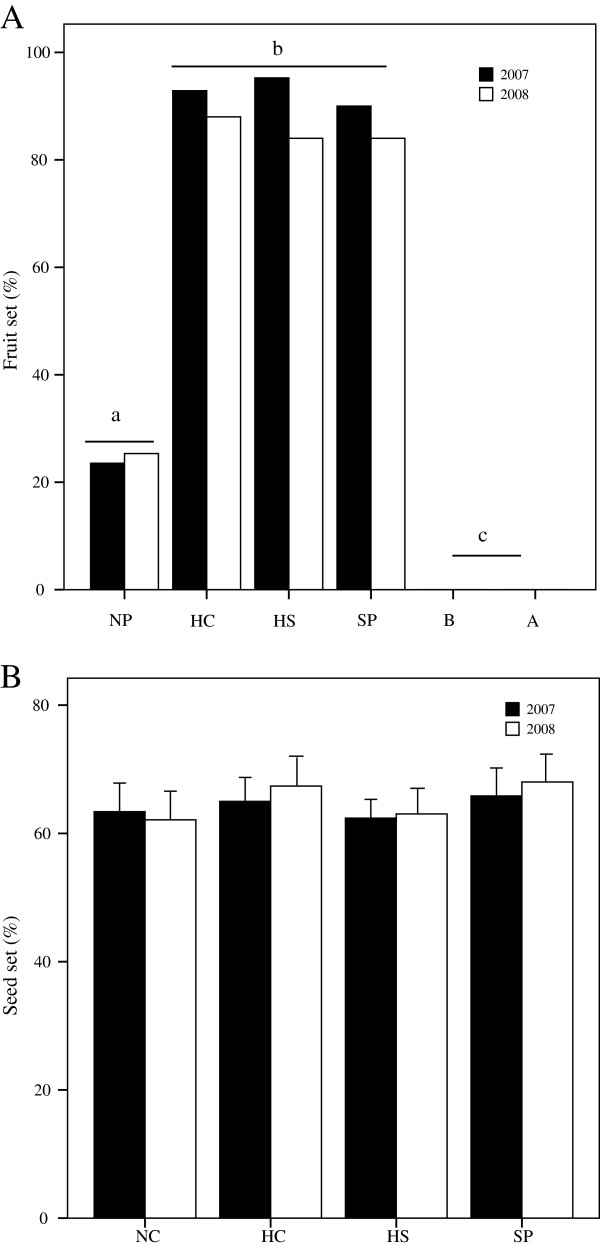
**Comparison of fruit set (A) and seed set (B) among pollination treatments in the population HLG.** NP, natural pollination; HC, hand cross-pollination; HS, hand self-pollination; SP, supplemented pollination; B, bagged, automatic self-pollination; A, apomixis. Bars with different letters differ statistically at *P* < 0.05.

Using a MANOVA, we found that pollination treatment had significant effects on female reproductive success (i.e. fruit set and seed set; Table 1). Natural fruit set (24.38%) was significantly lower than fruit set for hand-selfed, hand-crossed, and pollen-supplemented treatments (*F* = 751.5, P < 0.001) (Table 1), and no significant differences among the latter three treatments themselves were observed (Figure 2A). However, seed set of natural pollination was not significantly different from that of hand pollination treatments (*F* = 0.591, *P* = 0.622) (Figure 2B, Table 1). These results indicated that *I. mairei* was highly self-compatible. Nevertheless, in both autonomous apomixis and autogamy treatments, none of flowers set fruit (N = 20) during either of the two years. Therefore, this species is incapable of autonomous apomixis and autogamous pollination, and is entirely dependent upon pollinators for female reproductive success.

**Table 1:** **Effect of pollination treatment (natural pollination, hand cross-pollination, hand self-pollination and supplemented pollination) and year (2007 and 2008) on levels of fruit set and seed set in *Incarvillea mairei***

**(a)**	**MANOVA (df = 2,157)**		**Fruit set (df = 1,158)**		**Seed set (df = 1,158)**	
**Factor**	**Wilks’λ**	***F***	**SS**	***F***	**SS**	***F***
Treatment	0.007	572.70**	12.539	751.5**	0.060	0.591
Year	0.456	91.95**	0.103	185.07**	0.004	0.11

Note: Asterisks indicate MANOVA or univariate ANOVAs that are statistically significant: **P < 0.001.

### Pollen limitation

**Figure 3:**
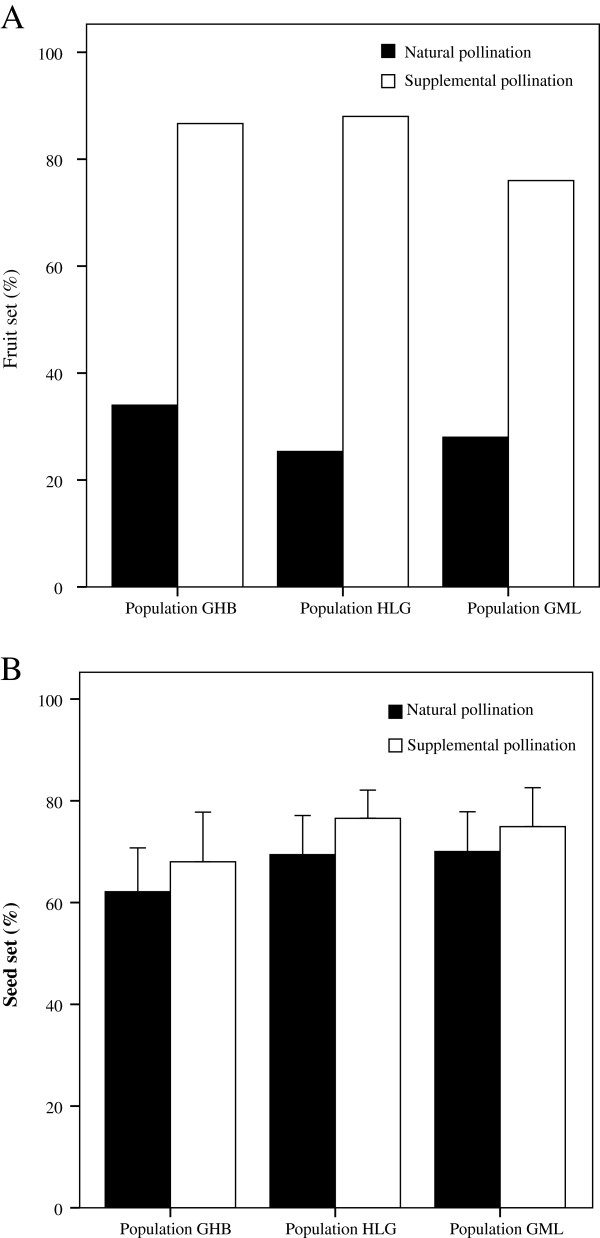
**Comparison of fruit (A) and seed set (B) between natural pollination and supplemental pollination treatments.** Bars with different letters differ statistically at *P* < 0.05. Black bars: natural pollination; grey bars: supplemental pollination.

The MANOVA and univariate ANOVA revealed that both pollination treatment and year significantly affected pollen limitation and reproductive output (Table 1 and Figure 2). In both 2007 and 2008, the supplemental pollination greatly increased the fruit set compared to natural pollination (*F* = 185.07, *P* < 0.001) (Figure 2A), although no significant differences in seed set were observed between natural and supplemental pollination in HLG (*F* = 0.11, *P* = 0.733) (Figure 2B). Next, the fruit and seed set of both natural and supplemental pollination were compared among the three populations in 2008 (Table 2 and Figure 3). Across all populations, the average fruit set from natural pollination was 26.3% (with a range of 24% ~ 28%), and the supplemental pollination significantly increased the fruit set, which was 83.8% and 3.2 times higher than from natural pollination (*F* = 918.6, *P* < 0.001) (Figure 3A). However, mean percentage seed set of natural pollinated flowers was high, averaging 67.2% and ranging from 62.1% to 70%, although no significant differences among populations (*F* = 1.152, *P* = 0.323). The mean percentage seed set was even higher for supplementarily pollinated flowers than for naturally pollinated flowers among three populations, but the difference was not statistically significant (*F* = 1.448, *P* = 0.485) (Figure 3B). Both the fruit and seed set were not significantly different among populations at different altitudes (*F* = 7.207, *P* = 0.063, and *F* = 2.486, *P* = 0.088, respectively) (Table 2 and Figure 3).

**Table 2:** **Effect of pollination treatment (supplemented hand pollination or control) and plot type (low, medium and high) on levels of fruit set and seed set in *Incarvillea mairei***

	**MANOVA (df = 2,117)**		**Fruit set (df = 1,118)**		**Seed set (df = 1,118)**	
**Factor**	**Wilks’λ**	***F***	**SS**	***F***	**SS**	***F***
Treatment	0.012	4.554**	8.893	918.6**	0.107	3.391
Population	0.428	30.438	0.014	7.207	0.157	2.486

Note: Asterisks indicate MANOVA or univariate ANOVAs that are statistically significant: **P < 0.001.

### Genetic diversity of adults

The seven analyzed microsatellites loci yielded 56 alleles with an effective number of 8 alleles per locus. The observed heterozygosity (*H*_O_) and the expected heterozygosity (*H*_E_) per locus ranged from 0.267 to 0.967 and from 0.242 to 0.881, respectively (Table 3). Fixation indices (F) at population level ranged from −0.119 to 0.115, while the inbreeding coefficients (F_IS_) varied from −0.502 to 0.225 per locus. Genetic diversity was not significantly different among the studied populations [mean *H*_O_ (SE) = 0.669 (0.060); mean *H*_E_ of 0.662 (0.047)].

**Table 3:** **Genetic diversity of seven microsatellite loci in adult plants from three populations of *Incarvillea mairei***

**Locus**	**GHB (N = 30)**			**HLG (N = 30)**			**GML (N = 30)**		
	***A***	***H***_**E**_	***H***_**O**_	***A***	***H***_**E**_	***H***_**O**_	***A***	***H***_**E**_	***H***_**O**_
IM6	4	0.521	0.767	6	0.642	0.733	2	0.508	0.967*
IM7	7	0.833	0.800	8	0.797	0.800	5	0.623	0.267*
IM26	6	0.674	0.400*	6	0.682	0.533	3	0.666	0.567*
IM48	7	0.781	0.833	8	0.776	0.633	8	0.820	0.767
IM73	10	0.881	0.933	6	0.770	0.733	4	0.242	0.267
IM75	4	0.644	0.600	4	0.691	0.333*	2	0.488	0.733*
IM78	5	0.753	0.633	8	0.820	0.667	7	0.847	0.833
Average	6.143	0.727	0.709	6.571	0.740	0.633	4.429	0.599	0.629

Note: A, number of allele per locus; *H*_E_, expected heterozygosity; *H*_O_, observed heterozygosity; *statistically significant deviation from Hardy-Weinberg equilibrium at *P* < 0.01.

### Outcrossing rate and pollen donor composition

Among the three natural populations, the outcrossing rate (*t*_*m*_) ranged from 0.970 to 0.988 (Table 4). Minimum estimates of apparent selfing due to biparental inbreeding (*t*_m_- *t*_s_) ranged from 0.058 to 0.180, with a mean value of 0.128 ± 0.136 among the 45 mother plants. The *t*_*m*_ - *t*_*s*_ value departed significantly from zero (at 95% confidence intervals), showing a significant amount of biparental inbreeding (Table 4).

**Table 4:** **Mating system parameters for *Incarvillea mairei *** **estimated in each mother individual, population and over all three study sites**

**Population**	**Sample size ( *n *)**	**Outcrossing rate**		**Biparental inbreeding (*t***_**m **_**– *t***_**s**_**)**
		**Multilocus (*t***_**m**_**)**	**Single locus (*t***_**s**_**)**	
GHB	150	0.970 ±0.085	0.829 ± 0.058	0.141 ± 0.073
HLG	150	0.988 ± 0.072	0.808 ± 0.071	0.180 ± 0.062
GML	150	0.978 ± 0.065	0.920 ± 0.046	0.058 ± 0.054

The average correlated paternity varied from 0.488 ± 0.086 in GML to 0.999 ± 0.164 in HLG with the Ritland mixed mating model, and from 0.432 ± 0.051 in GML to 0.602 ± 0.026 in HLG using TWOGENER (Table 5). The effective number of pollen donors, which is the reciprocal of correlated paternity, varied from 1.001 in HLG to 2.049 in GML using the Ritland mixed mating model and from 1.661 in HLG to 2.314 in GML using TWOGENER (Table 5).

**Table 5:** The estimates of correlated paternity (mean ± SE) and the effective number of pollen donors (the reciprocal of correlated paternity) extracted from Ritland mixed mating model and TWOGENER analysis

**Population**	**Ritland mixed mating model**		**TWOGENER analysis**	
	***r***_**p**_	**1/*r***_**p**_	**2Φ**_**FT**_	**1/2Φ**_**FT**_
GHB	0.491 ±0.105	2.036	0.470 ± 0.019	2.127
HLG	0.999 ± 0.164	1.001	0.602 ± 0.026	1.661
GML	0.488 ± 0.086	2.049	0.432 ± 0.051	2.314

## Discussion

### Self-compatiblity

Most of the species in the tropical family Bignoniaceae are trees, liana and shrubs, while only three groups have adopted a herbaceous habit. Since these groups occur mostly at high elevations in the Himalayas (*Incarvillea*) and the high Andes of South America (*Argylia*, *Tourrettia*) [40], it seems likely that evolution of herbaceous habit has evolved independently in these two regions in response to the common environment. The current distribution of *Incarvillea* was largely shaped by the uplift of the Himalayas [27]. Until now, only 38 of the approximately 800 species of Bignoniaceae have been studied in terms of reproductive biology, and 31 of these species have been found to be self-incompatible [31,41,42]. Our study revealed that *I. mairei* is fully self-compatible, and this result is consistent with recent studies that have found two congeners (*I. sinensis* and *I. arguta*) to both be self-compatible [29,30]. Owing to the shift from self-incompatibility to self-compatibility in these species, it would be of interest to determine the mating system in *Argylia* and *Tourrettia*, two other herbaceous genera of high altitude Andean in the Bignoniaceae.

### Pollinator limitation and floral longevity

There is a consensus that alpine plants are faced with severe pollinator restriction [2], which may reduce the possibilities for cross-pollination [3]. In response, these plants generally ensure reproductive success by increasing self-compatibility, apomixis, and vegetative reproduction [43,44]. On the other hand, pollen limitation is less intense in self-compatible species, compared to obligate outcrossing species [45]. Under such circumstances, pollinator unpredictability could lead to selection for the capacity for a combination of both selfing and outcrossing pollination strategies [7]. In this study, the fruit set of *I. mairei* flowers exposed to natural pollination (20-30%) was much lower than flowers receiving supplemental pollen (>80%) and the average value for self-compatible plants (72.5%) [46]. However, among flowers setting fruit, seed set was not significantly different between open- and hand-pollinated (Figure 3B). This suggests that fruit set failure in natural populations were mainly due to pollinator absence, rather than to insufficient pollen deposition during visitation.

The increased floral longevity in alpine plants is one evolutionary strategy employed to overcome sparse or unpredictable pollinator service in order to receive outcross pollen [1,17,25,47]. *I. mairei* with the high mountain habitats (altitude between 3000 and 4500 m) was one of the earliest flowering plants in the study sites, starting to flower shortly after snowmelt in early May and continuing to late June. Compared with *I. mairei*, the other two species studied have different habitats, i.e. *I. arguta* grown in dry and hot valley (1300-2700 m), while *I. sinensis* occurred in northern China, at low altitude (1100-1300 m), whose flowers last for 1–3 d and 5–9 h, respectively, flower longevity of *I. mairei* was extraordinarily long, ranging from 7 to 12 d under natural conditions. Furthermore, pollinator exclusion greatly increased floral longevity of *I. mairei*. A recent study showed that flowers with longer exposure times had a greater opportunity to be visited by pollinators and higher reproductive success than flowers with experimentally shortened exposure times [19]. In *I. mairei*, the pollinator-responsive flexibility of longevity of the large colorful flowers might be adaptive to unpredictable pollination conditions. In addition, we found that the number of flowers decreases with increasing altitudes, in the higher altitude population (GML), most individual plants have single flower, which may tend to reduce geitonogamous selfing, i.e., transfer of pollen between flowers of the same plant.

### Floral traits that promote outcrossing

*Incarvillea mairei* employs a predominantly outcrossing mating system according to parentage analysis (*t*_m_ > 0.9). Our study showed that outcrossing rates were high in all three high-altitude populations sampled. It seems that pollinator limitation could be of little importance in constraining seed set in *I. mairei*, as supplemental pollination did not enhance seed production in individual flowers compared to natural pollination. Furthermore, these results demonstrated that every seed family was nearly resulted from one or two pollinator visits (one in HLG; two in GHB and GML), a result consistent with very low visitation rates but high pollination efficiency. These results suggest that pollination of *I. mairei* is an “all or nothing” phenomenon, and this species may have evolved floral traits that maximize fitness from single pollinator visit to cope with low densities of pollinator and stochastic pollination.

In addition to longevity, other features of the floral biology of *I. mairei* seem to be well suited to counter pollen limitation. In Bignoniaceae, permanent stigmatic closure is common, especially resulting from the deposition of self-incompatible pollen [48,49]. The stigma in *I. mairei* is sensitive to touch by the pollinator. The closure of stigma in *I. mairei* (6.86 ± 3.06 s) is quicker than in other members of the Bignonicaceae (eg, *Jacaranda rugosa*[49] and *Campsis radicans*[48] stigmas normally close >40s after being touched). Stigma closure in *I. mairei* usually occurred rapidly once the pollinator visited the flower, and maintained complete closure before the pollinator left. Therefore, the closure of stigma in *I. mairei* may effectively prevent self-pollination, and this is supported by our field observations of the pollination process and the high outcrossing rates. Besides, stigma closure reduced pollen loss from stigma [50] that might result during the common rain events in the Himalayan summer.

The stigma in *I. mairei* reopened if receiving insufficient pollen, regardless of whether self- or cross-pollen. As suggested by Fetscher [51], reopening of stigma in *I. mairei* could allow a second opportunity for the receipt of pollen that could fertilize additional ovules. This function would be especially adaptive in populations where seed production is limited even after two visits (ie, GHB and GML).

The pollen-dispensing efficiency with respect to pollen load was promoted by its unique anther morphology during the foraging phase of visitation. In *I. mairei*, each anther has a spur that protrudes at a right-angle to the anther surface, downwards into the corolla tube. Our observations indicate that the anther dehiscence and pollen dispersal were triggered when pollinators stimulate the opposite anther spur. The interaction of spur-pollinator would largely minimize the waste of pollen and enhance male function [29]. On the other hand, plant fitness via male function would appear to be limited in this species owing to the low rate of pollinator visitation.

## Conclusions

The floral biology of *I. mairei* consists of a suite of mechanisms which appear to promote high pollination efficiency despite the low availability of pollinators. The combination of the movement of sensitive stigmas, anther prongs, and pollinator behavior a remarkably efficient breeding system, and make it possible to ensure successful reproduction in the harsh alpine environment. Recognition of this mechanism may be of great significance to our understanding the adaptation, speciation and diversity of alpine plants in the Himalaya-Hengduan mountains region.

## Competing interest

The authors declare that they have no competing interests.

## Authors’ contributions

HA, HW and DL designed this study. HA performed the experiments. WZ and HA analyzed the data. WZ and KX contributed reagents/materials/analysis tools. HA, WZ, HW and DL wrote the manuscript. All authors read and approved the final manuscript.
